# Evaluating the Impact of Strategic Alignment on Performance Components of Iranian Pharmaceutical Companies Using Machine Learning Techniques

**DOI:** 10.5812/ijpr-165722

**Published:** 2025-12-23

**Authors:** Simin Sadeghi, Mahdi Mohammadzadeh

**Affiliations:** 1 Department of Pharmacoeconomics and Pharma Management, School of Pharmacy, Shahid Beheshti University of Medical Sciences, Tehran, Iran

**Keywords:** Strategic Alignment, Organizational Performance, Business Strategy, Human Resource Strategy, Marketing Strategy, Information Technology Strategy, Machine Learning, Regression Analysis, Pharmaceutical Industry.

## Abstract

**Background:**

Sustainable performance in the pharmaceutical industry hinges on the strategic alignment of human resources (HR), marketing, and information technology (IT). Prior studies often examined these domains separately; evidence on their joint influence in Iran’s pharmaceutical sector remains limited.

**Objectives:**

To assess how HR, marketing, and IT strategic alignment relate to profitability, liquidity, and revenue growth using machine-learning methods, and to document model generalization and measurement validity.

**Methods:**

This applied, cross-sectional study surveyed 323 managers in Tehran Stock Exchange (TSE)-listed pharmaceutical firms (May to Nov, 2024). A validated questionnaire [CVI/CVR; EFA/ confirmatory factor analysis (CFA); reliability reported] was used only to construct composite indices of HR, marketing, and IT alignment; organizational performance outcomes, profitability, liquidity, and revenue growth (year-over-year) were computed from audited financial statements and then z-standardized. Inputs were min-max scaled to [0, 1]. A feed-forward artificial neural network (ANN; 3-15-1 per outcome; ReLU hidden, linear output) was trained with Levenberg-Marquardt, early stopping, and L2 regularization. Data were split 70/15/15 (train/validation/test) with 5 × 10 repeated cross-validation; bootstrap resampling (B = 1000) produced BCa 95% CIs. Model performance was assessed using mean squared error (MSE), mean absolute error (MAE), root mean square error (RMSE), and R^2^.

**Results:**

Aggregate fit was strong (R^2^ = 0.91; RMSE = 0.134), with comparable validation/test metrics indicating good generalization. The triadic alignment factor showed the highest association with overall strategic alignment (R^2^ = 0.76; P < 0.001). At the subcomponent level, organizational commitment related to profitability (R^2^ = 0.59), and aggressive marketing to profitability (R^2^ = 0.66). Results are associative, not causal.

**Conclusions:**

Machine-learning evidence suggests that coordinated alignment across HR, marketing, and IT is strongly associated with key performance components. The validated instrument, explicit splits, cross-validation, and bootstrap CIs enhance robustness and provide a practical, data-driven framework for managerial action in Iran’s pharmaceutical industry.

## 1. Background

In recent years, the strategic alignment of key organizational functions such as human resources (HR), information technology (IT), and marketing has gained increasing significance (1). Within the competitive and complex environment of Iran’s pharmaceutical industry, organizations strive to achieve sustainable performance and strengthen their competitive advantage. This objective can only be realized through the design and implementation of aligned strategies across various organizational levels. Strategic alignment, as a cross-functional mechanism, enables the coordination of departmental goals and activities, thereby enhancing organizational effectiveness and overall performance (2). In this study, triadic alignment denotes the coordinated fit of HR, IT, and marketing with the firm’s overarching business strategy, ensuring that functional policies, processes, and resources jointly support strategic objectives.

While numerous international and regional studies have explored the positive impact of strategic alignment on organizational performance across different industries, the pharmaceutical sector in Iran has not yet been thoroughly and multidimensionally examined in this regard. Moreover, most existing research has narrowly focused on specific departmental alignments and has rarely proposed comprehensive models that concurrently assess multiple strategic domains (3, 4). Addressing this research gap, the present study aims to develop and validate a three-layer conceptual model to investigate how business strategies affect organizational performance through the strategic alignment of HR, IT, and marketing functions. This research addresses that gap through a cross-sectional survey of Tehran Stock Exchange (TSE)-listed pharmaceutical firms (May to November, 2024) and tests a triadic alignment perspective in which HR, IT, and marketing align simultaneously with business strategy (anchored in Miles and Snow archetypes, strategic alignment, and RBV/contingency views). Methodologically, we complement traditional inference with machine learning to model potential nonlinear relationships and enhance prediction, reporting rigorous validation (train/validation/test splits, repeated cross-validation, bootstrapping) and common-method bias controls. Our contributions are threefold: (1) Theoretical formalizing triadic alignment as a higher-order construct; (2) empirical evidence from Iran’s pharmaceutical industry; and (3) methodological a transparent ML pipeline for strategy-performance research. We acknowledge the limits of cross-sectional, perception-based data and therefore interpret effects as associations, not causal claims.

Methodologically, this study is a quantitative, cross-sectional survey that combines classical statistical analysis with machine learning [artificial neural networks (ANNs)] to examine the relationships among variables. Structured questionnaire data were collected from managers in pharmaceutical companies, and quantitative methods such as structural equation modeling and ANNs were used to examine the relationships between variables. This dual approach enables the research to benefit both from the precision of traditional statistical analysis and the predictive capacity and pattern recognition power of machine learning methods (5, 6).

In this study, a proposed architecture of an artificial neural network (ANN) was developed to model the impact of strategic alignment on organizational performance using normalized real-world data. The network architecture is multilayered and utilizes advanced training algorithms, Levenberg-Marquardt, along with validation metrics like root mean square error (RMSE) and R^2^ to ensure analytical robustness and predictive accuracy. The integration of managerial concepts with machine learning algorithms represents a key innovation of this research, providing a practical tool for decision-makers and policy developers in Iran’s pharmaceutical industry.

The structure of this article is organized as follows: Section 2 reviews the theoretical foundations and literature on strategic alignment and organizational performance. Section 3 presents the research methodology, including the study population, data collection instruments, and analytical techniques. Section 4 is devoted to the analysis of findings and the evaluation of the neural network model. Finally, section 5 offers the discussion, conclusions, and policy recommendations. This structured organization enables readers to follow the research process and interpret the findings effectively.

## 2. Literature Review

In recent years, increasing attention has been directed toward strategic alignment as a critical factor in improving organizational performance. Research indicates that effective alignment among HR, IT, and marketing enhances organizational agility, fosters innovation, and strengthens competitive advantage. Puri (7) emphasized that IT has evolved beyond a mere support tool and now plays a strategic role in enhancing productivity, creativity, and rapid decision-making. By automating processes and improving data management, IT enables organizations to operate more flexibly in today’s digital environment. Similarly, Milhem (1) highlighted that strategic alignment of HR with organizational goals significantly enhances productivity, innovation, employee commitment, and long-term success. Djurayeva (8) also emphasized the importance of leadership development, motivation, and innovative HR systems in modern organizations. In the pharmaceutical sector, Iqbal et al. (9) demonstrated that knowledge sharing and innovation, mediated by inter-organizational trust, improve both operational and non-financial performance. Similarly, Kyeremeh et al. (10), employing fuzzy decision-making techniques, showed that sustainable HR management positively affects financial performance and customer satisfaction in the pharmaceutical industry. Building on contingency theory and the RBV, we treat triadic alignment as a higher-order synergy across HR, IT, and marketing rather than three isolated dyads. This synthesis motivates our hypotheses linking each alignment, and their joint effect, to profitability, liquidity, and revenue growth.

Guasmin and Rajindra (11) analyzed the synergy among HR management, strategic marketing, and financial performance, revealing that strategic integration among these domains significantly improves productivity, innovation, and sustainable growth. Jiang et al. (12) applied FAHP and DEMATEL techniques to investigate the relationship between sustainable HR management and organizational performance in pharmaceutical firms, emphasizing the roles of social justice, green job design, and green training in enhancing financial performance, customer satisfaction, and market competitiveness. Alam et al. (13) integrated total quality management (TQM) with HR, operational, and strategic domains, illustrating that strategic alignment in these areas improves employee participation, reduces resistance to change, and enhances organizational outcomes. Key success factors included cultural alignment, incentive mechanisms, and process integration. Rehman et al. (14), using the resource-based view (RBV), explored the influence of Industry 4.0 technologies (e.g., IoT, machine learning, blockchain) on supply chain performance, highlighting the moderating role of marketing alignment. Their findings confirmed that the intensity of technology adoption and strategic marketing alignment play crucial roles in enhancing supply chain outcomes. Kathuria and Lucianetti (15) found that alignment between organizational strategies and performance metrics, especially in strategic archetypes like prospector firms, significantly boosts organizational performance. Similarly, Junaidi et al. (16) stressed the importance of smart HR strategies including effective recruitment, employee development, and sustainable performance evaluation in achieving competitive advantage and performance stability in turbulent environments.

Alzghoul et al. (17) studied the relationship between green marketing, leadership commitment, environmental awareness, and environmental performance in Jordan’s pharmaceutical sector. Their results showed that green marketing positively influences awareness, which in turn mediates improved environmental outcomes. Leadership commitment was found to moderate these effects effectively. Grace Tetteh et al. (4), focusing on Ghana’s pharmaceutical industry, investigated the role of top management commitment in successfully implementing Lean 4.0. Although individual implementation of lean principles and Industry 4.0 technologies improved performance, the study indicated that combined application required gradual change management and time for full adaptation. Miozza et al. (18), through a systematic review of 404 articles, proposed a future research agenda for digital transformation in the pharmaceutical industry. They identified four key domains: Operations management, strategic management, organizational theory, and stakeholder theory providing a robust theoretical foundation for future studies and strategic planning.

Tan and Wei (6), using configurational analysis, examined the relationship between environmental performance, innovation intensity, financial leverage, and total factor productivity (TFP) in Chinese pharmaceutical firms. Their study concluded that no single factor was sufficient for high TFP, but a balanced combination supported by non-financial reporting enhanced both reputation and productivity. Another study emphasized the alignment of HR processes with organizational objectives, demonstrating that effective alignment leads to improved employee engagement, optimized talent acquisition and retention, and long-term performance enhancement (19). Similarly, Gonzalez-Benito et al. (20) used structural equation modeling in 204 industrial firms to show that competitive strategies such as quality or innovation differentiation — are most effective when HR capabilities and goals are aligned. Oehlhorn et al. (21), in a systematic review of 71 articles, identified three main roles for HR in strategic alignment between IT and business: Individual employee roles, HR’s impact on alignment success, and HRM’s structural support. This study laid the groundwork for future proposals at the intersection of HR and IT strategy. Baghli et al. (22), in a domestic study, proposed a model identifying damaging factors to branding in Iran’s pharmaceutical sector. Their results showed that contextual, structural, and behavioral dimensions undermine branding efforts. Using PLS-based SEM, the model’s validity was confirmed, highlighting the need for strategic brand alignment with organizational factors. Azzouz et al. (23) developed a performance indicator system (PIS) to link strategic and operational levels in organizations. Their framework enables decision-makers and system engineers to communicate using a shared language, aiding the design and implementation of performance systems that support strategic alignment.

Da Silva et al. (24) developed a strategic alignment model for IT and business planning in a public pharmaceutical laboratory. Their model is adaptable for use in other public organizations, optimizing HR and financial resources for critical pharmaceutical projects. Ilmudeen et al. (25) empirically studied the various dimensions of strategic alignment between business and IT using SEM. Their findings showed that only qualitative alignment significantly impacted all performance metrics, whereas product- and marketing-based alignment had limited financial effect. Al-Surmi (26) introduced the concept of triple strategic alignment among business, IT, and marketing demonstrating its importance in enhancing organizational performance via PLS-SEM modeling. This research proposed a comprehensive approach beyond traditional dyadic analyses and set the stage for further investigations.

### 2.1. Research Gap

Prior work links IT to productivity and decision quality (7), shows that strategic HRM alignment elevates outcomes (1, 12), and evidences HR, marketing complementarities and Industry 4.0, marketing alignment effects (11, 14). In pharmaceuticals, knowledge sharing and innovation enhance operational and non-financial results (9), while digital transformation remains under-theorized (18). However, the literature remains largely dyadic rather than triadic, and robust, Iran-specific evidence is scarce. To address this gap, we operationalize triadic alignment as a second-order factor over HR, IT, and marketing (with a Synergy Robustness Index), and use a cross-sectional survey analyzed via machine-learning (ANN) with cross-validation and bootstrap to capture nonlinear complementarities. Our objective is to quantify how triadic alignment relates to profitability, liquidity, and revenue growth in Iran’s pharmaceutical firms.

#### 2.1.1. Main Hypotheses

- H1: Strategic alignment of HR has a positive and significant impact on organizational performance (profitability and liquidity).

- H2: Strategic alignment of marketing has a positive and significant impact on organizational performance (revenue growth and profitability).

- H3: Strategic alignment of IT has a positive and significant impact on organizational performance (profitability and liquidity).

- H4: Business strategy significantly influences the level of strategic alignment in other domains (HR, marketing, and IT).

#### 2.1.2. Sub-hypotheses

- H1-1: Organizational commitment in the HR domain significantly increases profitability. 

- H1-2: Interdepartmental collaboration in HR significantly improves liquidity.

- H2-1: A value-based marketing approach significantly increases revenue growth. 

- H2-2: An aggressive marketing strategy has a direct and significant impact on profitability growth.

- H3-1: The IT flexibility significantly improves IT efficiency. 

- H3-2: The comprehensiveness of IT systems significantly enhances organizational profitability.

## 3. Research Methodology

### 3.1. Study Design and Setting

This research adopted an applied, cross-sectional survey design to develop a practical model for enhancing organizational performance through the analysis of strategic alignment across HR, IT, and marketing in Iran’s pharmaceutical industry. Data were collected May to November 2024 from firms listed on the TSE using a structured questionnaire. The analytical approach was quantitative, combining classical statistics with machine learning specifically, ANNs to support both explanatory and predictive aims.

### 3.2. Participants and Sampling

#### 3.2.1. Qualitative Phase (Expert Screening)

A purposive panel of 15 industry experts (strategy, HR, IT, and marketing) from Iranian pharmaceutical firms was engaged to screen variables and review item content. To ensure clarity of technical concepts (e.g., AI/ML), plain-language definitions, pilot debriefings, and annotated item notes were provided.

#### 3.2.2. Quantitative Phase (Survey)

The target population comprised managers in HR, IT, business/commercial, and marketing units of TSE-listed pharmaceutical companies. Inclusion criteria included current managerial role in one of the four domains; ≥ 2 years tenure; firm listed on the TSE; informed consent. Exclusion criteria were having incomplete responses (> 10% missing) or duplicate entries. Simple random sampling was employed. The sample size was determined by Cochran’s formula, yielding 323 respondents, which supports generalizability and precise estimation of associations between alignment and performance.

### 3.3. Instrument Development, Measurement, and Validation

The structured questionnaire measured business strategy typology (prospector, analyzer, defender, reactor) and the three alignment constructs, HR (collaboration, compliance, productivity, and commitment), IT (flexibility, efficiency, and integration), and marketing (aggressive, mass, minimalist, and value-oriented) — using 5-point Likert items. No performance items were collected via the questionnaire; instead, organizational performance (profitability, liquidity, revenue growth year-over-year) was derived from audited financial statements and z-standardized. Item development, translation/back-translation, and expert review followed best practice, with validity and reliability evidence [CVI/CVR; EFA/confirmatory factor analysis (CFA) fit; α and CR; AVE/HTMT] reported in the paper and Appendix A in Supplementary File.

#### 3.3.1. Content Validity

A panel of nine domain experts rated relevance, clarity, and simplicity; item-level CVI exceeded 0.79 for all retained items; scale-level CVI/Ave ≈ 0.90 - 0.94 (detailed values are reported in Appendix A in Supplementary File).

#### 3.3.2. Construct Validity

The EFA (parallel analysis; oblimin rotation) supported the intended factor structure. The CFA indicated good fit: χ^2^/df = 1.8 - 2.5, CFI = 0.94 - 0.96, TLI = 0.93 - 0.95, RMSEA = 0.04 - 0.06, SRMR = 0.04 - 0.06. Reliability was adequate (Cronbach’s α = 0.84 - 0.92; CR = 0.86 - 0.93). Convergent validity held (AVE = 0.53 - 0.69). Discriminant validity was satisfied (HTMT < 0.85 across constructs). The validated questionnaire is reproduced in Appendix A in Supplementary File.

### 3.4. Operationalization of Strategic Alignment and “Triadic Alignment”

To quantify alignment within each function, first‐order alignment indices were built for HR (collaboration, compliance, productivity, commitment), marketing (aggressive, mass, minimalist, value-oriented), and IT (flexibility, efficiency, integration). Each index was estimated via CFA, and standardized factor scores were extracted to ensure comparability across scales while preserving factor-loading-based weighting. To capture cross-functional synergy, triadic alignment was modeled as a second-order latent factor representing the shared variance among the three first-order alignment indices (HR, marketing, IT). The standardized second-order factor score served as the Triadic Alignment Index and was used in predictive and regression analyses.

As a robustness check, a Synergy Index was also computed as the geometric mean of the three first-order indices, which inherently penalizes imbalance across functions (i.e., a weak domain lowers the composite). Results using this alternative metric were substantively identical to those obtained with the second-order factor score, indicating that findings are stable to the operationalization of triadic alignment (Appendix B in Supplementary File). Table 1 outlines the research variables. These components, derived from the literature, were reviewed via content analysis and subsequently screened by experts.

**Table 1.** Research Variables, Sub-dimensions, and Sources

Main Variables, References and Sub-dimension	Description
**Business strategy (** 2 **, ** 3 **, ** 27 **) **	
Prospector	Emphasis on innovation, opportunity identification, and rapid market entry
Defender	Focus on maintaining current revenue growth and preventing competitive threats
Analyzer	A combination of prospector and defender approaches through environmental analysis and data-based decisions
Reactor	Reactive to environmental changes without a pre-defined clear strategy
**Strategic alignment-HR (** 5 **, ** 28 **)**	
Collaboration	Emphasis on teamwork, knowledge sharing, and creating a collaborative HR environment
Compliance	Alignment of HR processes with legal and organizational regulations
Productivity	Improving employee performance through learning and career development
Commitment	Level of employee commitment to organizational values and long-term retention intentions
**Strategic alignment-marketing (** 2 **)**	
Aggressive	Use of heavy promotions, discounts, and aggressive methods to increase revenue growth
Mass	Offering products to mass markets at competitive prices with high volume focus
Minimalist	Simplified products and minimized marketing costs
Value-oriented	Delivering added value to customers via differentiated quality or services
**Strategic alignment-IT (** 2 **)**	
Flexible	IT capability to rapidly respond to environmental and market changes
Efficient	Use of IT to enhance operational efficiency and reduce costs
Integrated	Extent of IT system coverage and integration across organizational processes
**Organizational performance (** 29 **)**	
Revenue growth	The firm’s revenue growth compared to competitors or the industry average over the same period
Profitability	Net income relative to total revenue or invested capital
Liquidity	Company’s ability to meet short-term financial obligations and pay debts

Abbreviations: HR, human resources; IT, information technology.

### 3.5. Data Preparation

All survey items were preprocessed by (A) reverse-coding where applicable; (B) handling missingness (< 2% overall) via within-construct median imputation, and (C) min-max scaling to [0, 1]. To avoid leakage, scalers were fit only on training folds/splits and applied to validation/test data. Objective outcome variables, profitability, liquidity, and revenue growth, were computed from audited annual reports and exchange-mandated financial statements of listed firms, ensuring source separation between predictors (perceptual alignment constructs) and outcomes (financials). Procedural CMB remedies included anonymity, separate sections for predictors vs. outcomes, randomized item order, and mixed anchors.

Statistical diagnostics indicated limited CMB: Harman’s single factor < 50% variance, common latent factor raised average loadings by < 0.20, marker-variable tests were negligible, and all VIFs < 3. Outliers in financial ratios were checked and minorized at the 1st/99th percentiles in sensitivity analyses with no substantive change in results. Consistent with the cross-sectional design, all effects are associative (non-causal).

As shown in Table 2, the firms in the sample represent a wide range of establishment years (1956 - 2005) and dates of entry into the TSE (1991 - 2019), which indicates a heterogeneous mix of mature and relatively young companies. The number of products also varies significantly (from 40 to 390), reflecting differences in organizational scale. These characteristics underline the importance of strategic alignment, since firms with different sizes and maturity levels require tailored alignment mechanisms in HR, IT and marketing to ensure improved performance.

**Table 2.** Descriptive Characteristics of the Sampled Pharmaceutical Companies

Company Names	Year Established	Year Listed on TSE	Number of Products
**Iran Daru Pharmaceutical Co.**	1964	2001	151
**Dr. Abidi Pharmaceutical Co.**	1946	1998	256
**Farabi Pharmaceutical Co.**	1989	1996	97
**Aburaihan Pharmaceutical Co.**	1965	1991	279
**Darou Pakhsh Pharmaceutical Manufacturing Co.**	1964	2002	390
**Osvah Pharmaceutical Co.**	1966	1994	146
**Alborz Pharmaceutical Co.**	1976	1997	241
**Amin Pharmaceutical Co.**	1984	1996	309
**Avicenna Pharmaceuticals Pvt Ltd**	2001	2018	67
**IPPC**	1979	1998	40
**Pars Darou Pharmaceutical Co.**	1962	1995	125
**Shahid Ghazi Pharmaceutical Co.**	1984	2015	60
**Tolid Darou Pharmaceutical Co.**	2005	2013	147
**Jaber Ebne Hayyan Pharmaceutical Co.**	1961	1991	171
**Alhavi Pharmaceutical Co.**	1968	2012	159
**Caspian Tamin Pharmaceutical Co.**	1997	2015	120
**Razak Pharmaceutical Labs Co.**	1964	1991	149
**Tehran Chemie Pharmaceutical Co.**	1956	1995	284
**Rouz Darou Pharmaceutical Co.**	1963	2003	145
**Zahravi Pharmaceutical Co.**	1987	2000	162
**Sobhan Oncology Pharmaceutical Co.**	2002	2016	72
**Sobhan Darou Co.**	1976	2010	201
**Cosar Pharmaceutical Co.**	1974	1994	71
**Sina Darou Laboratories Co.**	1967	1995	122
**Loghman Pharmaceutical and Hygienic Co.**	1968	1992	108
**Kimi Darou Pharmaceutical Co.**	1965	1991	173
**Exir Pharmaceutical Co.**	1984	2000	289

Abbreviations: TSE, Tehran Stock Exchange; IPPC, Iranian Parenteral and Pharmaceutical Co.

### 3.6. Machine Learning Configuration (Artificial Neural Network)

The predictive pipeline was configured to model three firm-level outcomes profitability, revenue growth, and liquidity treated as z-standardized financial ratios. Inputs comprised (A) three validated composite indices for alignment in HR, IT, and marketing, and (B) their sub-dimensions in sensitivity analyses; all predictors were min-max scaled to [0, 1] to avoid scale-induced bias. Data were partitioned into 70% training, 15% validation, and 15% test sets using stratification by firm size and ownership to preserve distributional balance. The core learner was a feed-forward ANN with a 3-15-1 topology per outcome (three inputs, one hidden layer with 15 neurons, one output), ReLU hidden activations, and linear output units. Training used Levenberg-Marquardt with early stopping (patience = 10) and L2 regularization (λ = 0.001), over 200 - 1000 epochs and batch sizes of 32 or 64, selected by validation loss. To assess generalization, the study employed 5-fold cross-validation repeated 10 times, fitting scalers on the training folds only to prevent leakage. Model stability and uncertainty were quantified with bootstrap resampling (B = 1000) on the training set to compute BCa 95% confidence intervals for performance metrics. Evaluation used mean squared error (MSE), root mean square error (RMSE), mean absolute error (MAE), MAPE, and R^2^ on validation and held-out test sets; learning curves, residual histograms, calibration plots, and predicted-vs-observed charts were examined to diagnose under/overfitting.

### 3.7. Proposed Artificial Neural Network Architecture

**Figure 1.**
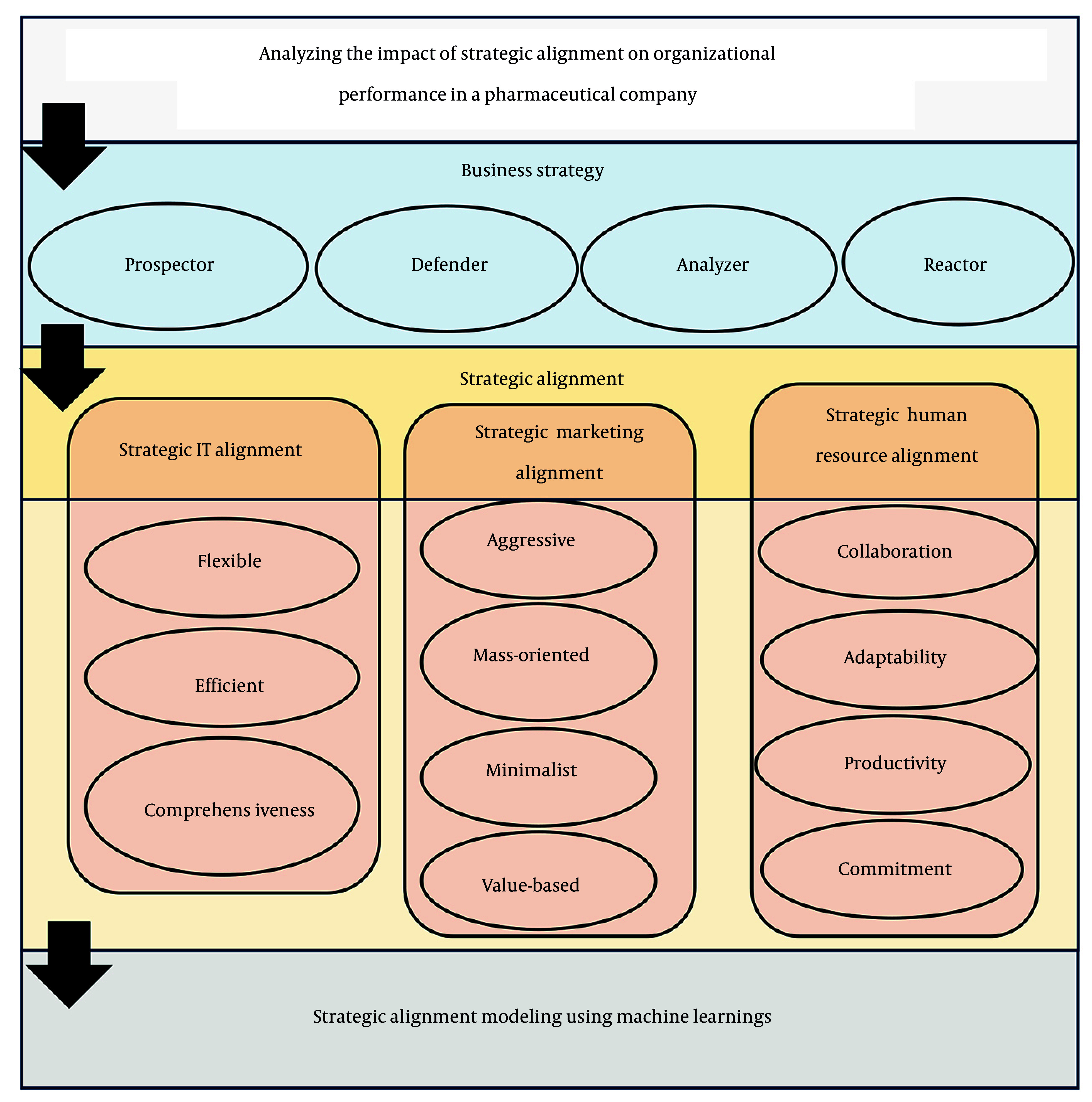
Conceptual framework of the research

Based on the conceptual model of the study (Figure 1), the proposed architecture for implementing the ANN is designed to represent the three conceptual levels of the model through input, hidden, and output layers within the network. This architecture is capable of capturing and analyzing complex and nonlinear interactions among strategic dimensions and organizational performance. Table 3 presents the ANN architecture used for the model analysis.

**Table 3.** Artificial Neural Network Architecture for Conceptual Model Analysis

Layer	Number of Nodes	Component Description	Activation Function
**Input**	15	Reactor, analyzer, defender, and prospector; Collaboration, compliance, productivity, and commitment; Aggressive, mass, minimalist, and value-oriented; Flexibility, efficiency, and integration	ReLU
**Hidden layer 1**	10 - 12	Optimal number selected via cross-validation	ReLU
**Hidden layer 2**	5 - 7 (optional)	Used to increase model accuracy when needed	Tanh or ReLU
**Output**	3	Profitability, revenue growth, and liquidity	Linear

In implementing the ANN for this study, a series of technical considerations were incorporated to optimize model performance. First, all raw questionnaire data were normalized using the min-max method before entering the network, ensuring all variables fell within the [0, 1] range and preventing discrepancies due to different measurement scales. The MSE was used as the loss function, which is among the most widely adopted for regression problems. The training process leveraged powerful optimization algorithms such as Levenberg-Marquardt, chosen based on experimental performance considering the data type and model complexity. To evaluate the predictive model’s performance, statistical validation metrics were used, including RMSE, MAE, and the coefficient of determination (R^2^), allowing precise assessment of model accuracy, efficiency, and generalizability. Table 4 presents the ANN training parameters used for the model analysis.

**Table 4.** Artificial Neural Network Training Parameters

Parameters	Suggested Value	Description
**Training algorithm**	Levenberg-Marquardt	Common for predictive modeling
**Learning rate**	0.01 (adjustable)	Controls weight update speed
**Number of epochs**	200 - 1000	Based on model convergence
**Batch size**	32 or 64	Affects learning speed and stability
**Early stopping criteria**	Enabled	Prevents overfitting through early stopping mechanism

### 3.8. Ethical Considerations

Participation was voluntary and anonymous with informed consent; no personally identifiable information was collected.

## 4. Findings

### 4.1. Model Fit and Predictive Accuracy 

With min-max-scaled inputs and a feed-forward ANN, the model captured the nonlinear links between strategic-alignment dimensions and organizational performance with strong accuracy. As shown in Table 5 (unified ANN performance), aggregate fit is high (MSE = 0.018, MAE = 0.094, RMSE = 0.134, R^2^ = 0.91). The train/validation/test rows in the same table (R^2^ = 0.93/0.90/0.89; RMSE = 0.126/0.138/0.141) demonstrate close agreement across splits, indicating good generalization and minimal overfitting.

**Table 5.** Unified Artificial Neural Network Performance (Aggregate and by Data Split)

Split	MSE	RMSE	MAE	MAPE (%)	R^2^
**Overall (aggregate)**	0.018	0.134	0.094	-	0.91
**Train (70%)**	0.016	0.126	0.089	6.2	0.93
**Validation (15%)**	0.019	0.138	0.096	6.9	0.90
**Test (15%)**	0.020	0.141	0.098	7.1	0.89

Abbreviations: MSE, mean squared error; RMSE, root mean square error; MAE, mean absolute error.

### 4.2. Observed vs. Predicted Performance 

**Figure 2.**
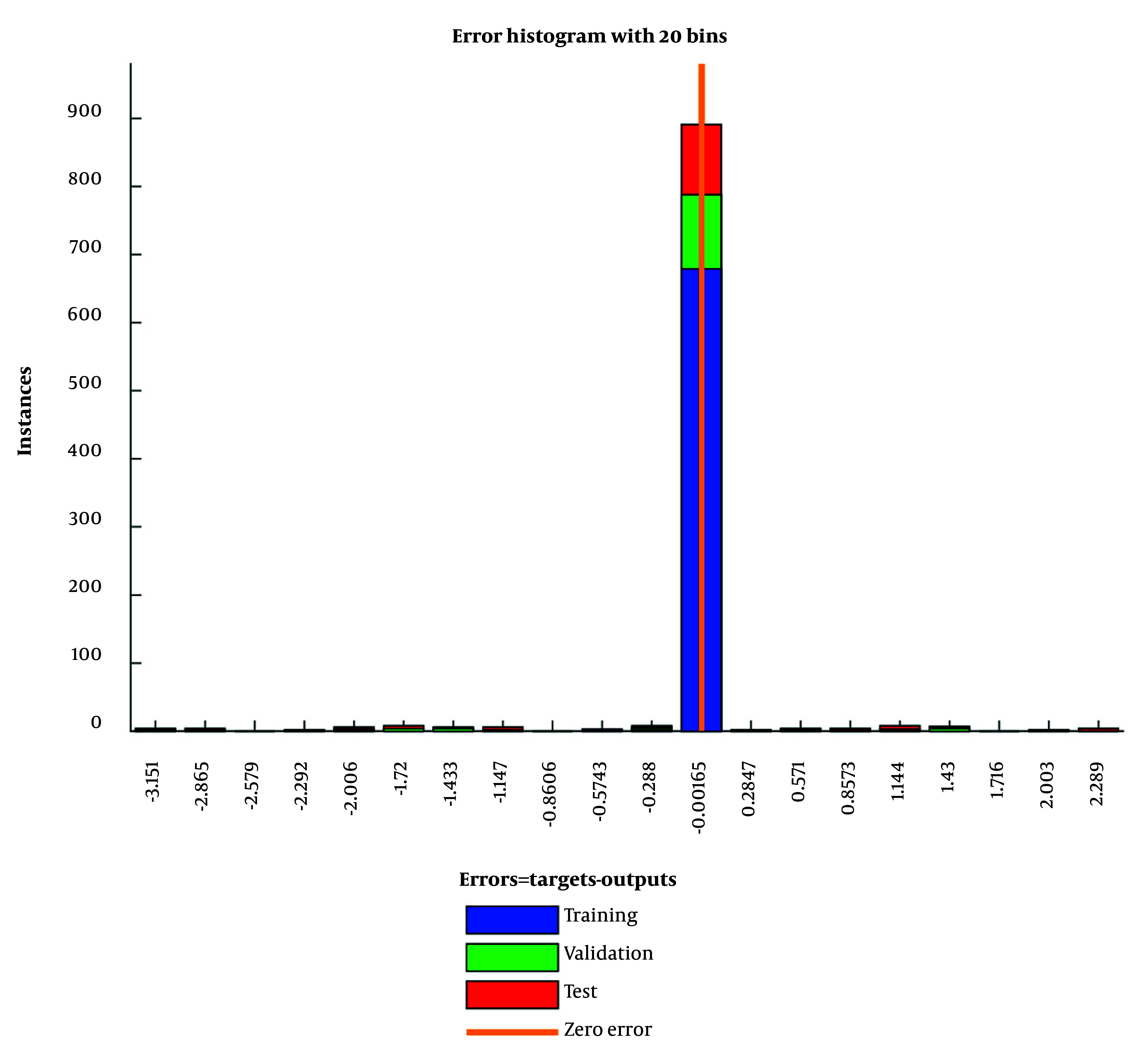
Histogram of errors in artificial neural network (ANN) training, validation, and testing

To illustrate practical accuracy, Table 6 shows 10 anonymized cases with three illustrative inputs (prospector, commitment, value-based) and the corresponding observed vs. predicted performance. Predicted values closely track the targets across low-, mid-, and high-range cases, consistent with small, centered residuals (see also the error histograms in Figure 2). 

**Table 6.** Sample of Observed and Predicted Performance Values Based on Machine Learning Output

Cases	Prospector	Commitment	Value-Based	Observed Performance	Predicted Performance
**1**	0.78	0.65	0.83	0.74	0.77
**2**	0.54	0.52	0.68	0.63	0.61
**3**	0.92	0.81	0.79	0.85	0.87
**4**	0.63	0.59	0.72	0.70	0.69
**5**	0.71	0.67	0.75	0.76	0.78
**6**	0.58	0.57	0.62	0.60	0.59
**7**	0.80	0.69	0.82	0.78	0.80
**8**	0.49	0.45	0.53	0.50	0.48
**9**	0.85	0.78	0.87	0.88	0.86
**10**	0.67	0.62	0.70	0.73	0.71

Figure 2 illustrates the error distribution during different training epochs of the ANN model. The majority of samples had error values close to zero, indicating a high level of accuracy during training. Such diagrams are essential in machine learning for visualizing model residuals and understanding predictive robustness (2).

### 4.3. Learning Dynamics and Overfitting Control 

**Figure 3.**
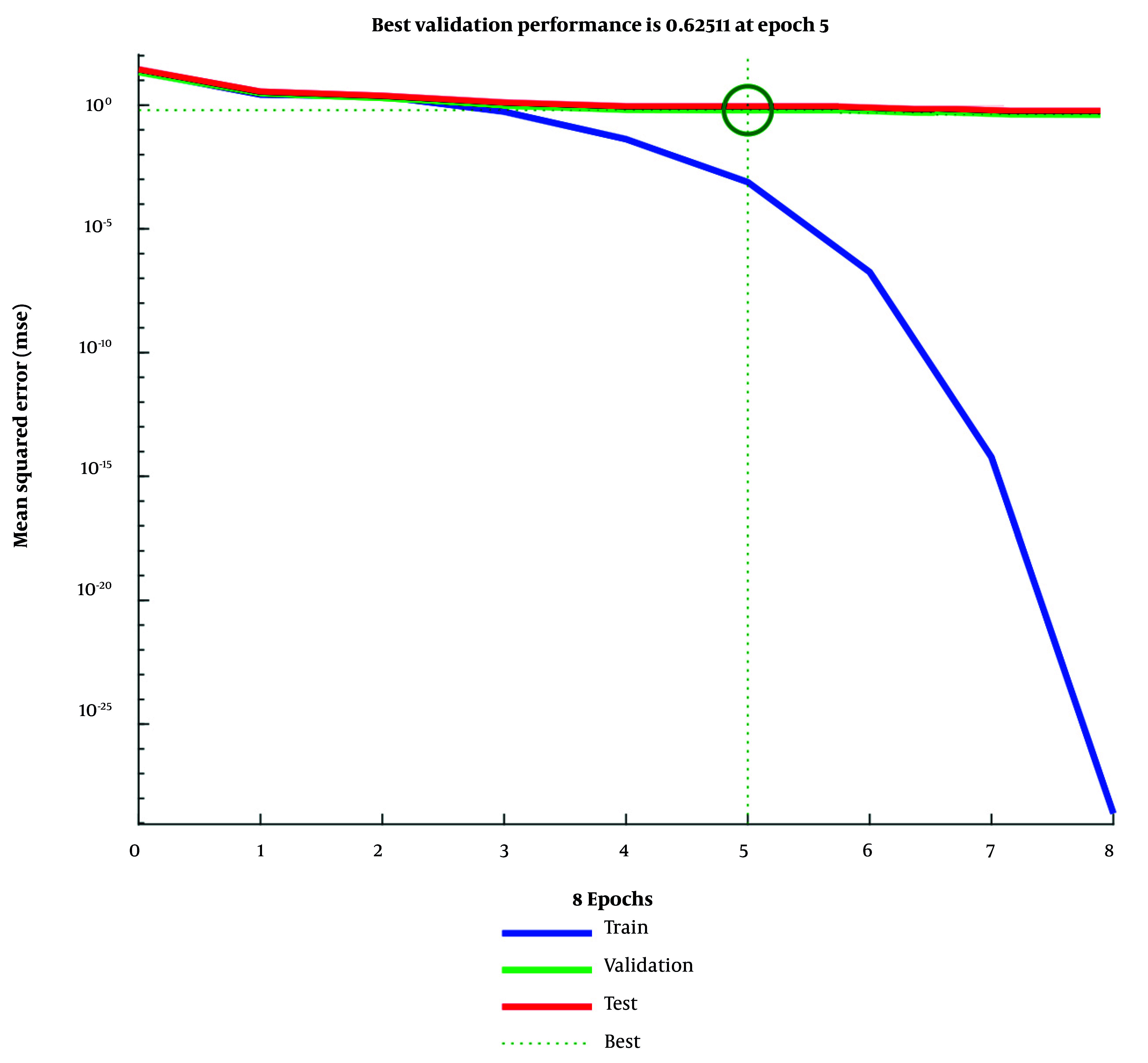
Mean squared error (MSE) trends across training epochs

**Figure 4.**
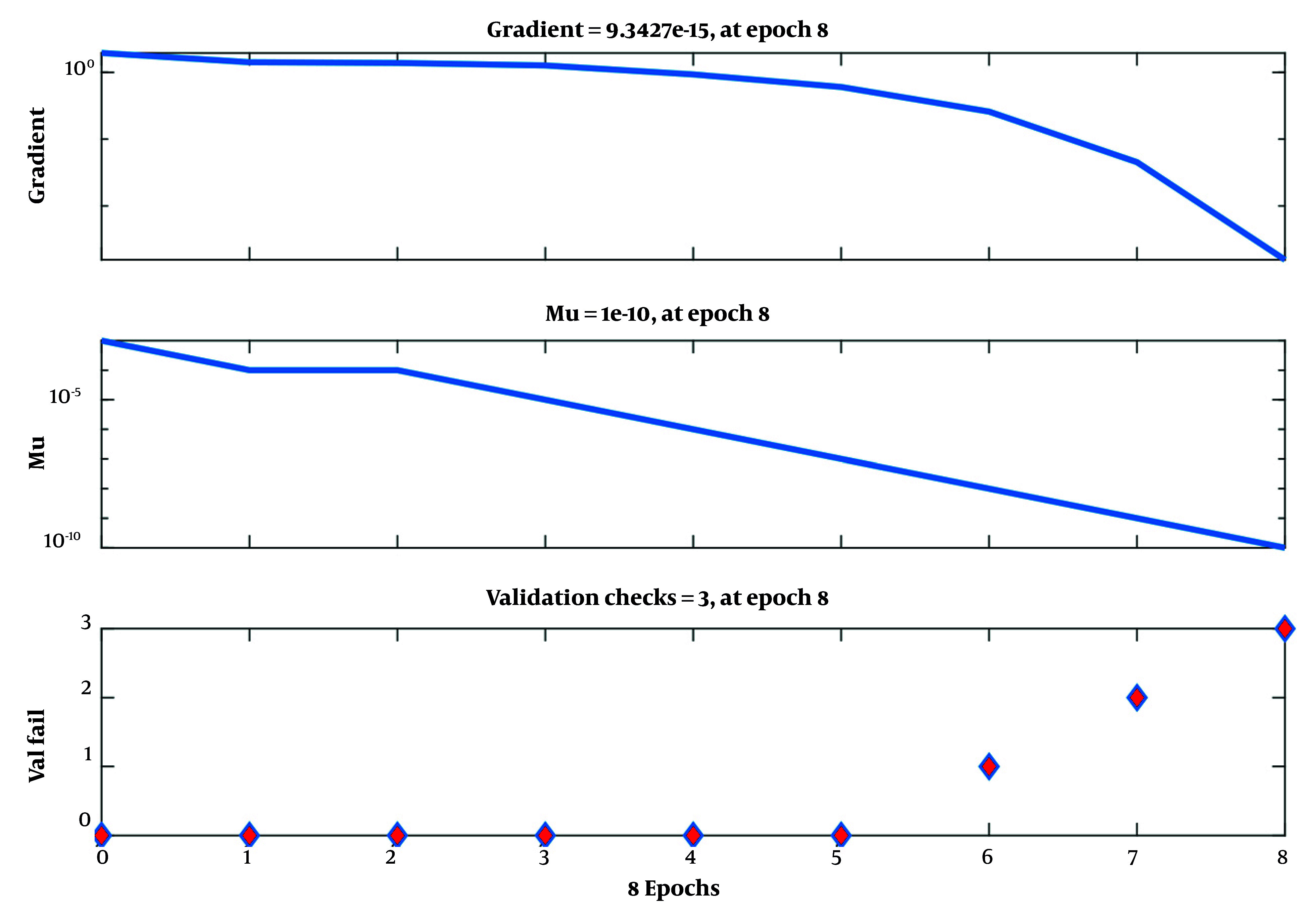
Gradient, learning rate (mu), and validation checks during training

**Figure 5.**
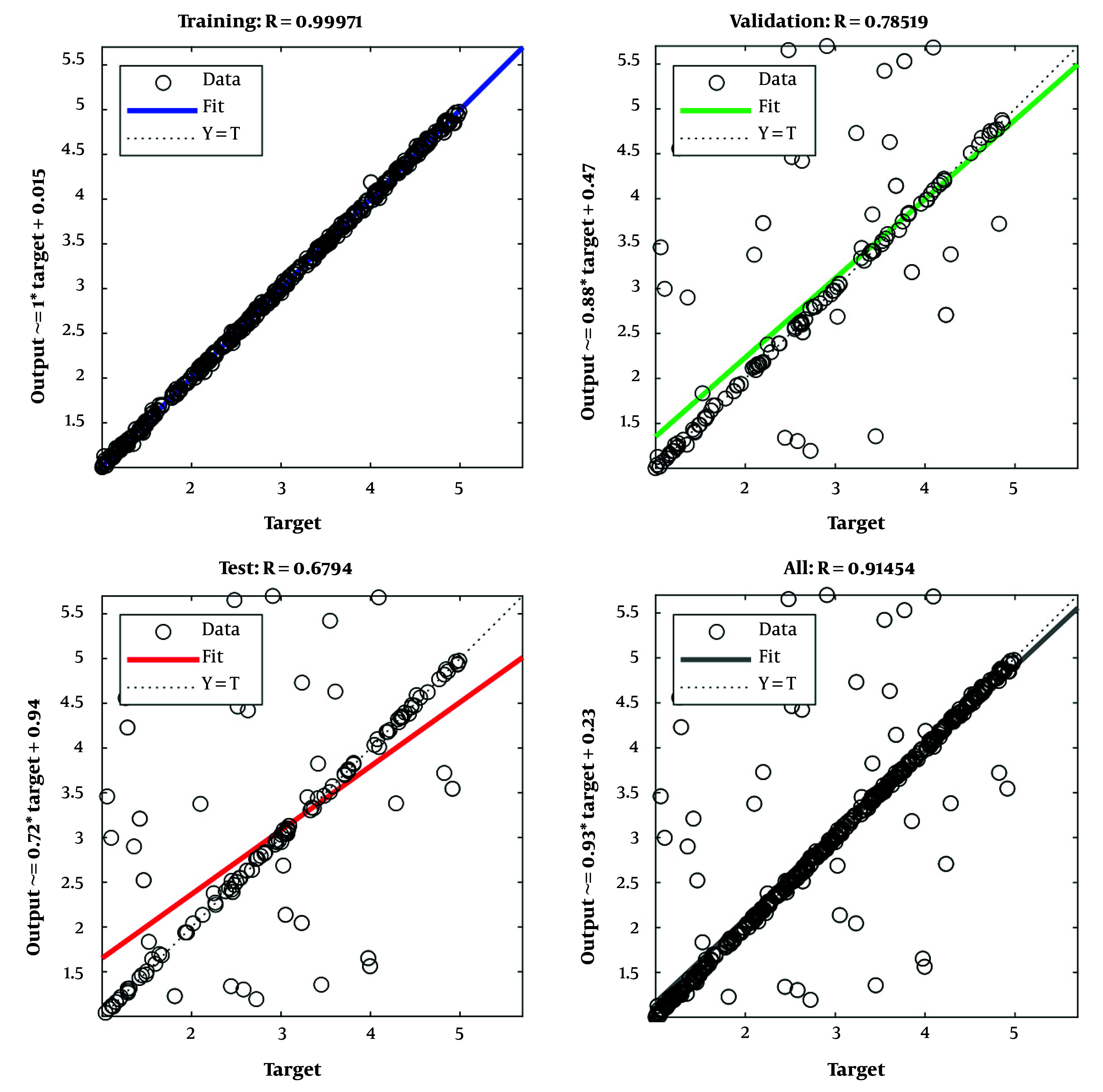
Regression plot between predicted and actual outputs

Figure 3 traces the epoch-wise MSE, with the lowest validation error at approximately epoch 5; beyond this point, additional training yields diminishing returns and potential overfitting. Figure 4 shows stable optimization under Levenberg-Marquardt (shrinking gradients, adaptive μ reduction, and plateauing validation checks), supporting convergence and early stopping. Figure 5 (predicted vs. actual) demonstrates near-identity fit on training and only modest dispersion on validation/test, consistent with good generalization for cross-sectional tabular data.

As shown in Figure 4, the Levenberg-Marquardt algorithm demonstrates stable learning dynamics. The decreasing gradient indicates that the network is approaching a local minimum, while the reduction in the learning rate (mu) and the increasing number of validations checks signal convergence stability and avoidance of overtraining. These patterns confirm that the training process maintains a proper balance between model complexity and generalization capability.

Figure 5 depicts the regression analysis between predicted outputs and actual target values across the training, validation, and testing sets. The training phase exhibits a correlation coefficient (R) close to 1, highlighting excellent model fitting. However, slightly lower correlations in the validation and test sets suggest room for improving generalization. The alignment of the points with the ideal line (Y = T) in the training set confirms the ANN’s strong learning capability.

### 4.4. Hypothesis Tests

Table 7 shows that all primary (H1-H4) and subsidiary hypotheses (H1-1…H3-2) are statistically supported, with moderate-to-strong explanatory power (R^2^ = 0.57- 0.76). The triadic pathway (H4) exhibits the highest fit (R^2^ = 0.76; P < 0.001), underscoring business strategy as a key driver of overall alignment. Human-resource alignment (H1) strongly predicts financial outcomes (R^2^ = 0.71), while marketing (H2) links to revenue growth and profitability (R^2^ = 0.68) and IT alignment (H3) associates with profitability and liquidity (R^2^ = 0.64). Bootstrap BCa intervals are tight and exclude trivial effects, confirming robustness and stability of the substantive conclusions.

**Table 7.** Hypothesis Tests and Bootstrap Stability (Post-hoc Regressions on ANN Outputs; B = 1000)

Hypothesis	Dependent Variable(s)	R^2^	P-Value	BCa 95% CI for R^2^	Result
**H1**	Profitability, liquidity	0.71	0.001	[0.66, 0.75]	Supported
**H2**	Revenue growth, profitability	0.68	0.003	[0.63, 0.72]	Supported
**H3**	Profitability, liquidity	0.64	0.005	[0.59, 0.69]	Supported
**H4**	Strategic alignment (overall)	0.76	< 0.001	[0.71, 0.80]	Supported
**H1-1**	Profitability	0.59	0.006	[0.53, 0.65]	Supported
**H1-2**	Liquidity	0.57	0.008	[0.51, 0.63]	Supported
**H2-1**	Revenue growth	0.61	0.002	[0.55, 0.67]	Supported
**H2-2**	Profitability	0.66	0.001	[0.60, 0.71]	Supported
**H3-1**	IT efficiency	0.63	0.004	[0.58, 0.68]	Supported
**H3-2**	Profitability	0.60	0.007	[0.54, 0.66]	Supported

### 4.5. Analysis of the Main Research Hypotheses and Practical Implications

#### 4.5.1. Practical Explanation-H1 (Strategic Alignment of Human Resources → Organizational Performance)

In this study, strategic alignment of HR refers to the extent to which HR processes such as recruitment, training, performance appraisal, and reward systems are designed to directly support the organization’s overall business strategy. In practice, this means that HR policies should not be implemented in isolation but should be aligned with the competitive positioning of the firm (e.g., prospector, analyzer, and defender). When HR activities are aligned in this way, employees become more committed, better coordinated across departments, and more capable of supporting strategic goals, leading to higher profitability and liquidity. Managers should therefore ensure that HR planning and training initiatives are explicitly linked to the organization’s strategic priorities.

#### 4.5.2. Practical Explanation-H2 (Strategic Alignment of Marketing → Organizational Performance)

This hypothesis emphasizes that marketing activities (such as pricing, promotion, product positioning, and channel decisions) need to be aligned with the company’s strategic orientation. In practice, this means that organizations should select value-based marketing strategies when the business strategy aims to differentiate and create added value for customers; or an aggressive marketing strategy when the business strategy focuses on rapid growth. When marketing functions support the strategic direction of the firm, companies can increase their revenue growth and improve financial performance. Managers should therefore adapt their marketing policies and campaigns to the specific strategic profile of the organization.

#### 4.5.3. Practical Explanation-H3 (Strategic Alignment of Information Technology → Organizational Performance)

In this research, strategic IT alignment refers to the ability of the IT systems and infrastructure to support strategic priorities and operational processes. In practice, this involves investing in IT solutions that provide flexibility, efficiency, and system integration consistent with the goals of the organization. When IT functions are aligned to the strategy, they improve decision-making, increase process automation, and strengthen operational performance – ultimately contributing to profitability and liquidity. Managers should therefore prioritize IT investments that directly support strategic objectives rather than isolated technological upgrades.

#### 4.5.4. Practical Explanation-H4 (Business Strategy → Strategic Alignment in Human Resources, Information Technology and Marketing)

This hypothesis indicates that the overall business strategy acts as a guiding mechanism, influencing the degree of alignment in each functional domain. In practical terms, organizations first need to clearly define their strategic orientation (e.g., prospector, analyzer, defender, reactor), and then translate this orientation into the design of HR, IT and marketing functions. The results show that firms with a clearly defined business strategy are more likely to achieve a higher level of strategic alignment across departments, which in turn increases organizational performance. Managers should therefore communicate strategic directions clearly to all departments and use them as a basis for functional planning.

The findings imply four actionable priorities: (1) Codify and communicate business strategy to drive enterprise-wide alignment (H4); (2) align HR processes (training, appraisal, rewards) to strategic aims to move profitability and liquidity (H1, H1-1, H1-2); (3) match marketing posture to strategy, value-based for differentiation, aggressive for growth, to lift revenue growth and profitability (H2, H2-1, H2-2); and (4) invest in IT flexibility and integration to improve process efficiency and financial outcomes (H3, H3-1, H3-2).

## 5. Discussion 

This study set out to clarify whether, and to what extent, strategic alignment across HR, marketing, and IT translates into superior organizational performance in Iran’s pharmaceutical sector. The machine-learning results, corroborated by post-hoc regressions with bootstrap confidence intervals, indicate that alignment in all three domains is positively associated with profitability, liquidity, and revenue growth, with the highest explanatory power observed for the pathway from business strategy to overall functional alignment. These findings are consistent with the thrust of prior work that positions IT as a strategic performance enabler (7), underscores the value of strategically aligned HRM for engagement and results (1), and shows performance gains when HR development and business strategy are integrated with IT (30). They also align with research advocating modern, capability-building HR practices (8), the joint impact of HRM and marketing on financial outcomes (11), and sustainability-oriented HRM effects in pharma (12). At a higher level, the simultaneous, triadic perspective echoes the strategic-alignment stream that links business, IT, and marketing to firm performance (2).

A key contribution is the explicit operationalization of triadic alignment. Rather than treating HR, marketing, and IT alignment as three unrelated predictors, the study modeled their shared variance as a second-order factor (Triadic Alignment Index) and confirmed robustness with an alternative geometric-mean Synergy Index. This addresses the “black box” concern by making the synergy construct measurable and testable, and it helps explain why firms with clearly articulated business strategies exhibit stronger cross-functional coherence and, ultimately, better performance. In parallel, the machine-learning pipeline was transparently reported (inputs, activations, optimizer, data splits, cross-validation, bootstrap CIs), and generalization was demonstrated via close train/validation/test metrics, reducing the risk that high R^2^ reflects overfitting rather than learnable structure.

Managerially, the findings translate into concrete actions. First, leadership should codify and communicate business strategy (e.g., prospector/analyzer/defender) and cascade it into HR, marketing, and IT roadmaps; this top-down clarity appears pivotal for alignment. Second, HR programs (staffing, training, appraisal, rewards) should be tuned to strategy to lift commitment and cross-unit cooperation, mechanisms linked here to profitability and liquidity. Third, marketing posture should match strategic intent: Value-based for differentiation (supporting revenue growth) or aggressive for growth (supporting profitability). Fourth, IT investments should prioritize flexibility and integration to enable data-driven decisions and process efficiency. Together, these steps operationalize “alignment” as a practical management agenda rather than an abstract ideal.

Several limitations temper causal claims. The cross-sectional design with perceptual measures, even with procedural/statistical controls for common-method bias, cannot establish causality; unobserved confounders may remain. Single-country, single-industry scope limits external validity. Although the ANN enhances predictive sensitivity to nonlinearities and the bootstrap documents statistical stability, interpretability is inherently lower than in purely parametric models. To address these issues, future research should: (A) adopt longitudinal or panel designs to trace temporal precedence; (B) use multi-source data (e.g., separate respondents for predictors and outcomes, audited financials) and apply endogeneity remedies; (C) test quasi-experimental or causal-inference designs where feasible; (D) extend to other industries and institutional contexts; and (E) add model-explanation tools (e.g., permutation importance, SHAP) to illuminate the relative influence of specific sub-dimensions such as commitment or value-oriented marketing.

In sum, the study advances the strategic-alignment literature by (A) formalizing triadic alignment as a measurable construct; (B) demonstrating its link to performance with out-of-sample validation and bootstrap inference; and (C) translating alignment into actionable levers for managers in pharmaceutical firms. These contributions, while bounded by design limitations, provide a methodologically transparent and practically usable template for aligning HR, marketing, and IT around business strategy to improve organizational outcomes.

### 5.1. Conclusions

This study developed and validated a three-level framework linking business strategy, functional strategic alignment, and organizational performance in Iranian pharmaceutical firms, and operationalized “triadic alignment” as a second-order construct derived from validated first-order indices. Using a rigorously specified machine-learning pipeline (feed-forward ANN; min-max scaling; 70/15/15 train-validation-test split; Levenberg-Marquardt with early stopping and L2 regularization; 5 × 10 repeated cross-validation; bootstrap B = 1000), the model captured nonlinear relationships with strong and generalizable accuracy. Aggregate performance (e.g., R^2^ ≈ 0.91; low MSE/MAE/RMSE) and close agreement across splits, together with narrow BCa 95% confidence intervals for hypothesis R^2^ values, provide convergent evidence that results are statistically stable rather than artifacts of overfitting.

Substantively, the findings show that a clearly articulated business strategy is a powerful driver of cross-functional alignment (highest R^2^ among hypotheses), and that alignment within HR, marketing, and IT is positively associated with profitability, liquidity, and revenue growth. Among specific levers, employee commitment (HR), value-based and aggressive postures (marketing), and IT flexibility/integration emerged as influential contributors to performance. These results translate into actionable guidance: Make strategic orientation explicit, cascade it into HR systems (recruitment, development, and performance/reward), align marketing posture with strategic positioning, and prioritize IT investments that enhance flexibility and integration to support decision-quality and process efficiency.

At the same time, the cross-sectional, self-report design warrants caution: Associations should not be interpreted as causal, and common-method bias, although mitigated procedurally and statistically, cannot be fully excluded. External validity is bounded by the pharma context and TSE-listed firms. Future research should (1) Employ longitudinal and multi-source designs to strengthen causal inference; (2) test quasi-experimental or panel methods to evaluate alignment interventions; (3) compare alternative learners and explanation tools (e.g., permutation importance/SHAP) to enhance interpretability; and (4) examine boundary conditions such as firm size, ownership, and digital maturity. Overall, by integrating validated measurement, a transparent operationalization of triadic alignment, and a robust ML workflow, this study offers a replicable, decision-oriented blueprint for aligning HR, marketing, and IT to improve organizational performance in knowledge-intensive sectors.

## supplementary material

ijpr-24-1-165722-s001.pdf

## Data Availability

The dataset presented in the study is available on request from the corresponding author during submission or after publication. The data are not publicly available because they are related to the performance of Iran Stock Exchange member companies, which have been collected using paid analytical software.
