# Supplementary material for: Evaluating the Impact of Strategic Alignment on Performance Components of Iranian Pharmaceutical Companies Using Machine Learning Techniques
Source: Iran J Pharm Res. 2025 Dec 23;24(1):e165722. doi: 10.5812/ijpr-165722 (PMC12915362; doi:10.5812/ijpr-165722)
Supplement: ijpr-24-1-165722-s001.pdf [file ijpr-24-1-165722-s001.pdf]

Appendix A. Instrument Validation

A1. Content Validity (CVI/CVR)

Construct	I-CVI (min–max)	S-CVI/Ave	CVR (avg)	Decision
Business Strategy	0.83–1.00	0.93	0.78	Retain
HR Alignment	0.81–1.00	0.92	0.76	Retain
Marketing Alignment	0.80–0.98	0.90	0.72	Retain
IT Alignment	0.82–1.00	0.91	0.74	Retain
Org Performance	0.84–1.00	0.94	0.80	Retain

(Panel: 9 experts; threshold I-CVI ≥ 0.79.)

A2. Construct Validity (EFA/CFA)

EFA: parallel analysis; oblimin rotation; factors load as theorized.

CFA fit (range across constructs): $\chi^2/df=1.8–2.5$; CFI=0.94–0.96; TLI=0.93–0.95; RMSEA=0.04–0.06; SRMR=0.04–0.06.

Reliability/Validity: $\alpha=0.84–0.92$; CR=0.86–0.93; AVE=0.53–0.69; HTMT<0.85.

Appendix B. Operationalization, Scoring, and Indices

B1. Pre-processing

Reverse code (if any), then Min–Max scale items to [0,1].

B2. First-Order Indices (factor-score approach)

For construct C with items x_j and CFA loadings λ_j:

$$\text{Index}_C = \frac{\sum_j \lambda_j \cdot z(x_j)}{\sum_j \lambda_j}$$

where $z(\cdot)$ are standardized inputs.

B3. Triadic Alignment (second-order factor)

Model a second-order latent TA over HR, IT, Marketing first-order indices; extract standardized second-order factor score as **Triadic Alignment Index**.

B4. Robustness (synergy) index

$$\text{Synergy} = (HR \times IT \times MK)^{1/3}$$

(geometric mean; penalizes imbalance). Results matched the second-order factor conclusions.

B5. Common-Method Bias Checks

Procedural: anonymity, separated sections, randomized order, mixed anchors.

Statistical: Harman's single factor < 50%; common latent factor Δloadings < 0.20; marker variable test negligible; all VIF < 3.

Appendix C. Questionnaire

Business Strategy

Proactiveness

-We constantly seek new opportunities related to the present operations

-We are usually, the first ones to introduce new brands or products in the market

-We constantly are on the lookout for businesses that can be acquired

Defensiveness

-We use cost control systems for monitoring performance

-We only produce a limited set of products where we have a competitive advantage over others

-We emphasize on product quality through the use of quality circles

Analysis

-We use IT to support strategic planning and decision-making

-When making a major decision, we usually try to develop thorough analysis

-We Emphasize effective coordination among different functional areas

IT: Information Technology Strategy

Flexibility

-We use IT for enhancing our ability to negotiate with our suppliers.

-We use IT for product marketing and promotion

-We use IT for obtaining customer feedback and providing service

Efficiency

-We use IT for support effective coordination across functions (e.g., marketing, manufacturing) and product lines.

-We use IT for improve the efficiency of our day-to-day business operations.

-We use IT to support manufacturing

Comprehensiveness

-We use IT to support strategic planning and decision-making

-We use IT in risk analysis of processes

-We use IT to forecast key indicators of business performance.

Marketing strategy

Aggressive Marketers

-We Develop innovative new products/services.

-We extensive marketing research to identify market segments with buyers that will pay premium prices.

-We Selective distribution through best distributors available.

-We Generate high-quality advertising materials.

-We use highly skilled and knowledgeable sales force for generate sales through internal sales force.

Mass Marketers

-We Offer a broad product/service line.
-We Use price promotions and discounts.
-We Evaluate salesperson performance based on achievement of targets or quotas.
-We Maintain high salesperson to sales manager ratio.

Marketing Minimizers

-We Provide products with a low probability of failure.
-Our Price is below industry average.
-We Distribute our products through an intensive distribution system.
-We Offer a focused product line.
-We offer premium value, high-quality products

Value Marketers

-We Regularly increase technical sophistication of products/services
-We Selective distribution through best distributors available.
-We Provide support to customer contact personnel
-We Evaluate salesperson performance based on accomplishment of prescribed behaviors.

HR: Human resources strategy

Commitment

-We recruit innovative employees with high technical standards.
-Our training activities for our employees are comprehensive, continuous, require extensive.
-In our company workers are encouraged to participate in decision-making

Productivity

-Performance appraisals for our employees are based on productivity and efficiency.
-Our training activities for our employees seek to increase short-term productivity.
-The recruitment/selection process for our employees involves screening many job candidates and is comprehensive (uses interviews, tests, etc.)

Compliance

-Our employees perform jobs that are well-defined
-Our training activities for our employees focus on compliance with rules, regulations, and procedures.
-Compensation/rewards for our employees focus primarily on their short-term performance.

Collaborative

-These employees perform jobs that require them to participate in cross-functional teams and networks.
-The recruitment/selection process for our employees emphasizes their ability to collaborate and work in teams.
-Performance appraisals for our employees are based on team performance
